# Qualitative and Quantitative Performance of Magnetic Resonance Image Compilation (MAGiC) Method: An Exploratory Analysis for Head and Neck Imaging

**DOI:** 10.3390/cancers14153624

**Published:** 2022-07-26

**Authors:** Amaresha Shridhar Konar, Ramesh Paudyal, Akash Deelip Shah, Maggie Fung, Suchandrima Banerjee, Abhay Dave, Nancy Lee, Vaios Hatzoglou, Amita Shukla-Dave

**Affiliations:** 1 Department of Medical Physics, Memorial Sloan Kettering Cancer Center, New York, NY 10065, USA; konarsha@mskcc.org (A.S.K.); paudyalr@mskcc.org (R.P.); 2 Department of Radiology, Memorial Sloan Kettering Cancer Center, New York, NY 10065, USA; shaha@mskcc.org (A.D.S.); hatzoglv@mskcc.org (V.H.); 3 General Electric Health Care, New York, NY 10065, USA; maggie.fung@med.ge.com (M.F.); suchandrima.banerjee@ge.com (S.B.); 4 Touro College of Osteopathic Medicine, New York, NY 10027, USA; adave3@student.touro.edu; 5 Department of Radiation Oncology, Memorial Sloan Kettering Cancer Center, New York, NY 10065, USA; leen2@mskcc.org

**Keywords:** synthetic MRI, relaxometry, MAGnetic resonance imaging Compilation (MAGiC), masseter muscle, head and neck (HN), qualitative, quantitative

## Abstract

**Simple Summary:**

Magnetic resonance imaging (MRI) is a widely used noninvasive imaging modality in the head and neck (HN) region to detect tumors. The new synthetic MRI method termed MAGnetic resonance imaging Compilation (MAGiC) uses a multi-dynamic multi-echo (MDME) sequence to generate quantitative T1 and T2 maps and multi-contrast qualitative images within a clinically feasible time. The testing of the synthetic MRI method was initially performed in the brain and subsequently extended to extracranial regions, including the prostate, breast, cervix, and rectum. The present exploratory study aims to investigate the performance of this new, rapid, synthetic MRI method for diagnostic image quality assessment and measurement of relaxometry metric values in HN tumors and normal-appearing masseter muscle. Initial results from 14 patients support the feasibility of generating synthetic T1w and T2w images from quantitative relaxometry mapping in the HN region.

**Abstract:**

The present exploratory study investigates the performance of a new, rapid, synthetic MRI method for diagnostic image quality assessment and measurement of relaxometry metric values in head and neck (HN) tumors and normal-appearing masseter muscle. The multi-dynamic multi-echo (MDME) sequence was used for data acquisition, followed by synthetic image reconstruction on a 3T MRI scanner for 14 patients (3 untreated and 11 treated). The MDME enables absolute quantification of physical tissue properties, including T1 and T2, with a shorter scan time than the current state-of-the-art methods used for relaxation measurements. The vendor termed the combined package MAGnetic resonance imaging Compilation (MAGiC). In total, 48 regions of interest (ROIs) were analyzed, drawn on normal-appearing masseter muscle and tumors in the HN region. Mean T1 and T2 values obtained from normal-appearing muscle were 880 ± 52 ms and 46 ± 3 ms, respectively. Mean T1 and T2 values obtained from tumors were 1930 ± 422 ms and 77 ± 13 ms, respectively, for the untreated group, 1745 ± 410 ms and 107 ± 61 ms, for the treated group. A total of 1552 images from both synthetic MRI and conventional clinical imaging were assessed by the radiologists to provide the rating for T1w and T2w image contrasts. The synthetically generated qualitative T2w images were acceptable and comparable to conventional diagnostic images (93% acceptability rating for both). The acceptability ratings for MAGiC-generated T1w, and conventional images were 64% and 100%, respectively. The benefit of MAGiC in HN imaging is twofold, providing relaxometry maps in a clinically feasible time and the ability to generate a different combination of contrast images in a single acquisition.

## 1. Introduction

Magnetic resonance imaging (MRI) is widely used for the noninvasive imaging of extracranial soft-tissue contrast in the head and neck (HN) region [1]. Technical challenges arise, due to patient movement, susceptibility artifacts, and the presence of metallic implants [2,3]. In routine radiological practice, HN MR imaging protocols are optimized specifically to the subsite, such as the oral cavity, oropharynx, nasopharynx, nasal cavity, larynx, neck, parotid, and thyroid. The qualitative high-resolution, multiplanar T1-weighted (T1w, spin–lattice relaxation) and T2-weighted (T2w, spin–spin relaxation) MR images are used to assess the location and extent of the HN tumors [1,4,5,6]. The quantitative measurement of relaxometry (T1 and T2 relaxation times) method offers the relationship between the MR images and tissue physiology. It has shown promising clinical applications in the brain including multiple sclerosis [7,8,9,10]. Previous studies have reported that the T1 and T2 metrics could be surrogate quantitative imaging biomarkers (QIBs) for free water content (cellularity) [11,12] and tumor morphology [13], respectively.

There is limited literature on quantitative MR relaxometry in the HN region. Watanabe et al. simultaneously quantified both the T1 and T2 values in the HN region, including the masseter muscle, using a mixed turbo-spin echo pulse sequence, which combines the principles of T1 weighting by inversion recovery and T2w by multi-echo sampling [14]. This method exhibited a variation in the tissue specific T1 and T2 values within different regions of the facial soft tissues [14]. There is still a need to quantify the T1 and T2 values for tumors in the HN region, which comprises a diverse group with a wide range of histological types. These can involve the pharynx, larynx, thyroid gland, sinonasal cavity, salivary glands, skull base, skin, and soft tissues of the HN [15,16]. 

The clinical utility of standard, quantitative T1, and T2 acquisition methods are inadequate, due to long scan times that make it unfeasible in radiological practice [17,18]. To overcome this challenge, Deoni et al. introduced a novel, rapid approach for separate high-resolution T1 and T2 relaxation mapping in the brain [17,19]. These advances in MRI relaxometry led to the development of a rapid, single time-efficient acquisition method. This approach was first initiated by Riederer et al., who proposed a synthetic MRI method to simultaneously quantify the T1 and T2 metric values [20]. Further refinements to this method were performed for brain imaging to generate quantitative T1 and T2 relaxation times, proton density (PD) maps, and adjustable qualitative T1w, T2w, and FLuid Attenuated Inversion Recovery (FLAIR) images in a single MRI acquisition [21,22,23]. General Electric Healthcare (GEHC, Waukesha, WI, USA), a major vendor, provide synthetic MRI, using multi-dynamic multi-echo (MDME) and termed it MAGiC (MAGnetic resonance imaging Compilation) [24]. The present study used this MAGiC method. 

Initial studies of MAGiC reported it to have comparable diagnostic image quality to conventional multi-contrast images in the brain [24,25]. This synthetic MRI method was subsequently extended to extracranial regions, including the prostate, breast, cervix, rectum, and spine [26,27,28,29,30,31,32]. For example, Zhao et al. reported the diagnostic image quality of synthetic and conventional images to be comparable in rectal cancer patients [30]. The same group also measured the quantitative T1 and T2 values, which have shown promise for determining the prognostic factors in rectal cancer [33]. 

To the best of our knowledge, the feasibility of generating T1 and T2 maps from synthetic MRI (MAGiC) has not yet been reported for tumors and masseter muscle in the HN region. The present exploratory study investigates the performance of this new, rapid, synthetic MRI method for diagnostic image quality assessment and the measurement of relaxometry metric values in HN tumors and normal-appearing masseter muscle.

## 2. Materials and Methods

### 2.1. Patient Selection

We followed institutional MRI guidelines for testing an MRI research prototype sequence that is not yet part of clinical HN diagnostic imaging. The Institutional Review Board approved this Health Insurance Portability and Accountability Act (HIPAA)-compliant prospective study for enrolled patients. Written informed consent was obtained from all of the eligible patients. The median age of the eligible 14 patients was 61 years (range, 29–83 years; eight males and six females), enrolled between October 2018 and August 2021. All of the eligible patients underwent conventional HN MR imaging with additional MAGiC sequence, irrespective of treatment timepoint (untreated (*n* = 3), treated (*n* = 11)). The exclusion criterion for patients was any contraindication to MRI, such as a noncompatible cardiac pacemaker. The patient characteristics, including treatment regime, are illustrated in Table 1.

### 2.2. MRI Data Acquisition

All of the patient examinations were performed on a 3T, 70 cm bore scanner (Discovery MR750w, General Electric Healthcare (GEHC, Waukesha, WI, USA), using an HN coil for imaging. The images were prospectively acquired, using a fixed set of scanning parameters and closely approximating the current standard-of-care for HN MR imaging protocols at the appropriate subsite. 

### 2.3. Standard Head and Neck Imaging

In this study, four different HN imaging protocols were used, depending on the subsite, for the fourteen patients as follows: neck (*n* = 2); nasal (*n* = 4); parotid (*n* = 6); nasopharynx (*n* = 2)). The standard clinical MR acquisition parameters were based on the specific HN imaging protocol by subsites. In general, all of the protocols included pre-contrast multiplanar (axial, coronal, and sagittal), T1w and T2w, fat-suppressed imaging with slice thickness (ST) ranging between 3–5 mm. Field of view (FOV) was between 18–22 cm, followed by post-contrast T1w imaging. For the T1w imaging, the range of TR = 600–800 ms, TE = minimum (~18 ms), and T2w imaging, TR = 3000–5000 ms, and TE = 100–102 ms.

### 2.4. Synthetic MR Imaging (MAGiC)

**Figure 1:**
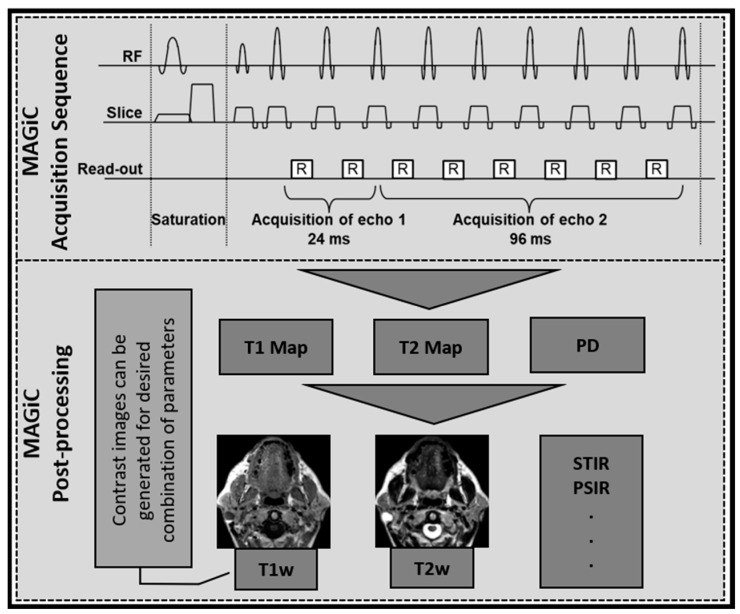
Synthetic MRI acquisition and post-processing: one representative block of MAGiC sequence, and MAGiC post-processing consist of generating quantitative and qualitative images. Qualitative images are generated using the relaxometry and proton density (PD) maps for the desired combination of parameters. Representative T1 and T2 images are shown, and additional contrast images can also be generated.

The MAGiC data for HN were acquired prior to contrast agent injection using MDME, a multi-dynamic multi-echo sequence. The MAGiC/MDME is based on the Quantification of Relaxation Times and Proton Density by Multiecho acquisition of a saturation-recovery using Turbo spin-Echo Readout (QRAPMASTER) sequence [22]. In QRAPMASTER, a continuous alteration of a slice-selective saturation and acquisition read-out results in a very efficient way of measuring T1, T2, and PD. Minor differences in the acquisition kernel exist for the standard MAGiC sequence, as available on the GE scanner, rather than the original Echo Planar Imaging (EPI) read-out and a composite echo image using multiple subsequent read-outs in QRAPMASTER, see Figure 1. QRAPMASTER had six echoes and an EPI read-out. MAGiC (MDME), available on the GE scanner, runs 10 or 12 echoes without EPI, but the echo read-outs are combined in the same k-space, resulting in only two effective echo images. Otherwise, the kernel is the same, with the continuous interleaved saturation and read-out. The 2-D axial MAGiC data were acquired with four automatically calculated saturation delays, two echo times (TE) (effective TE 24 and 96 ms), and a fixed TR of 4000 ms. The additional parameters were as follows: FA = 90°; Echo Train Length (ETL) = 12; ST = 5 mm; FOV = 20 cm; acquisition matrix 320 × 256; matrix reconstructed to 256 × 256. The total scan time was approximately 6 min. This produced four dynamics (saturation delays) and two echoes (MDME), resulting in eight different combinations. Each of these was saved as complex, real and imaginary images. Together, we had 16 images per slice.

### 2.5. MRI Data Processing

MAGiC, provided by GEHC, is a combined package with a data acquisition sequence and a post-processing pipeline that generates quantitative maps of T1 and T2 relaxation times. The contrast-weighted image settings can be defined on the post-process screen or the MAGiC session application. MAGiC was primarily developed for the brain where multiple contrast-weighted images, such as T1, T2, T1 FLAIR, T2 FLAIR, STIR, phase-sensitive inversion recovery (PSIR), and double inversion recovery (DIR) images, could be synthesized, using the quantitative maps for any desired combination of TE, TR, and TI. The MAGiC post-processing pipeline is shown in Figure 1. We synthetically generated T1w images using TR = 500 ms, TE = 10 ms, and T2w images with TR = 4500 ms, and TE = 100 ms. The focus of this exploratory study was to estimate the quantitative T1 and T2 maps and synthetically generated T1w and T2w images, as they are the most clinically relevant image contrasts. 

### 2.6. Regions of Interest Delineation

The regions of interest (ROIs) were delineated by an experienced neuroradiologist on the HN MAGiC data obtained from the 14 patients. To determine the extent of the tumor, anatomical T2w and post-contrast T1w images were used. The synthetic MRI simultaneously provides the T1 and T2 maps, and the ROIs were drawn on the T1 maps and translated to the T2 maps. In total, 48 ROIs were analyzed in this study, with the T1 and T2 maps combined. A total of 28 ROIs were from normal-appearing masseter muscle and 20 from tumors. 

For the tumor ROIs on the T1 and T2 maps, a single slice with the largest tumor dimension was used in the analysis. Out of the three untreated patients at the time of MRI, evaluable tumors were observed in two (four tumor ROIs on combined T1 and T2 maps), while one patient’s tumor size was below the inclusion threshold of 5 mm. For the 11 patients who underwent treatment, only 5 had 8 evaluable tumors (three patients had more than one tumor) with 16 tumor ROIs on combined T1 and T2 maps. Out of 11 treated patients, 6 showed a favorable response after treatment with no tumor visible at the time of the MRI.

### 2.7. Radiological Assessment

The diagnostic quality of both the synthetically generated (MAGiC) and the conventional clinical T1w and T2w images were assessed by an experienced neuroradiologist on standard workstations for image reading. The overall diagnostic image quality was rated (considering all of the available contrast views) on a five-point scale as follows: 5 = excellent (acceptable for diagnostic use); 4 = good (acceptable for diagnostic use); 3 = acceptable (acceptable for diagnostic use but with minor issues); 2 = poor (not acceptable for diagnostic use); or 1 = unacceptable (not acceptable for diagnostic use). Ratings of ≥3 were considered acceptable overall. 

For each contrast view, the reader rated the legibility (or the visibility of margins and structures associated with key anatomic/morphologic features) of anatomies defined a priori. The legibility ratings supplemented the overall image quality data, which considered six anatomical regions and provided specific information about the anatomic regions in HN imaging. Each anatomy was rated on a binary scale (legible/illegible), including the following: the submandibular gland; nasopharynx; the base of the tongue; sternocleidomastoid muscle; mandible; and glottis. The reader also recorded whether any of the following factors were seen in the images: low signal-to-noise; motion; wrap-around; contrast-to-noise; low image resolution; or blurring. The neuroradiologists evaluated a total of 1532 images to provide the rating for each contrast type (T1w and T2w) to compare between synthetically generated and conventional clinical images obtained from 14 patients. 

## 3. Results

**Figure 2:**
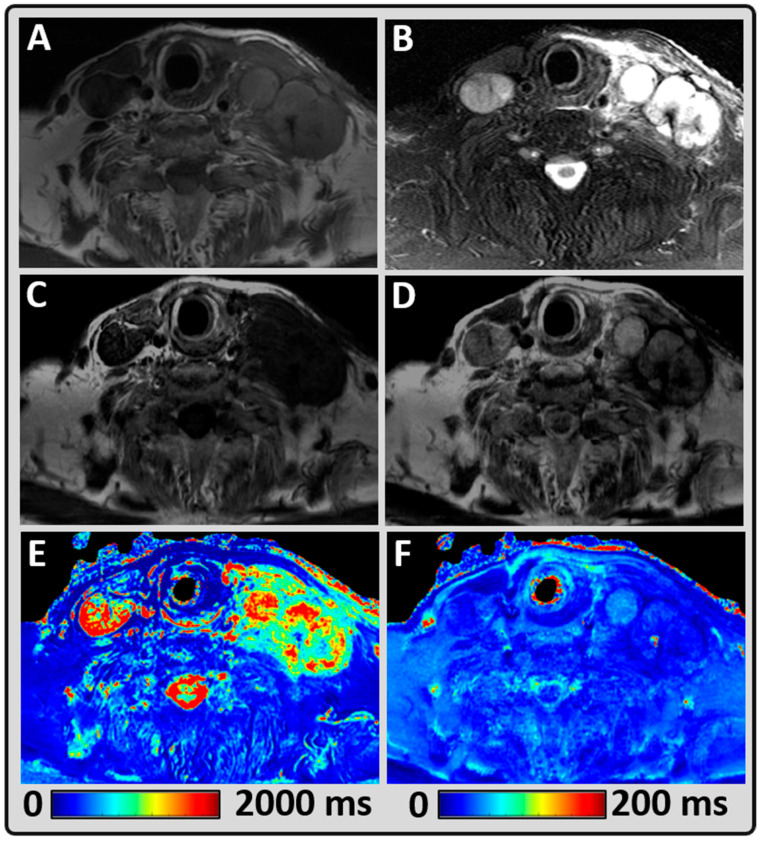
Representative MRI data from a 76-year-old male patient post-thyroidectomy with a bilateral metastatic supraclavicular lymph node. (**A**,**B**) Conventional qualitative T1- and T2-weighted (w) images depicting the metastatic tumors; (**C**,**D**) Synthetically generated T1w and T2w images using MAGiC; (**E**,**F**) MAGiC-generated quantitative T1 and T2 maps.

Figure 2 represents a patient’s data showing the synthetically generated qualitative images and quantitative relaxometry maps from the HN region. The mean T1 and T2 values measured within the metastatic neck node were 1905 ms and 88 ms, respectively. The qualitative diagnostic rating score for conventional T1w and T2w images was acceptable and good (rated three and four), respectively. The synthetically generated T1w and T2w images were both rated acceptable (3). The MAGiC sequence is unable to detect the long T2 structure, which is evident at the top right corner of the image and around the spine.

**Figure 3:**
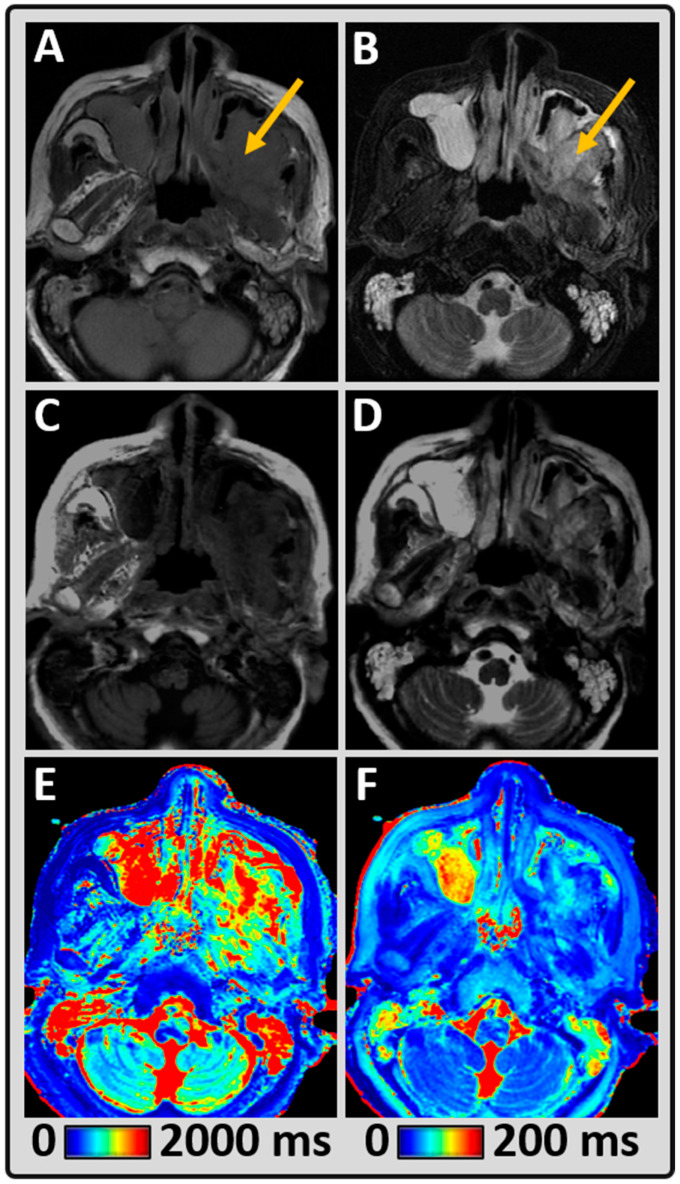
Representative MRI data from an 83-year-old female patient with yellow arrow pointing at left maxillary sinus secretory carcinoma of the salivary gland treated with radiation therapy. (**A,B**) Conventional qualitative T1w and T2w images demonstrating the tumor lesion; (**C,D**) Synthetically generated T1w and T2w images using MAGiC; (**E**,**F**) MAGiC-generated quantitative T1 and T2 maps.

Figure 3 shows the second representative HN patient data and shows a heterogeneously enhancing mass, centered in the left maxillary sinus. The mean T1 and T2 values measured within the tumor were 1571 ms and 75 ms, respectively. The qualitative diagnostic rating score for the conventional T1 and T2w images was good and poor, respectively (rated four and two). The synthetically generated T1w and T2w were both acceptable (rated three). In this example, the diagnostic T2w images were poor, while the synthetically generated T2w images were clinically useful and added value.

**Figure 4:**
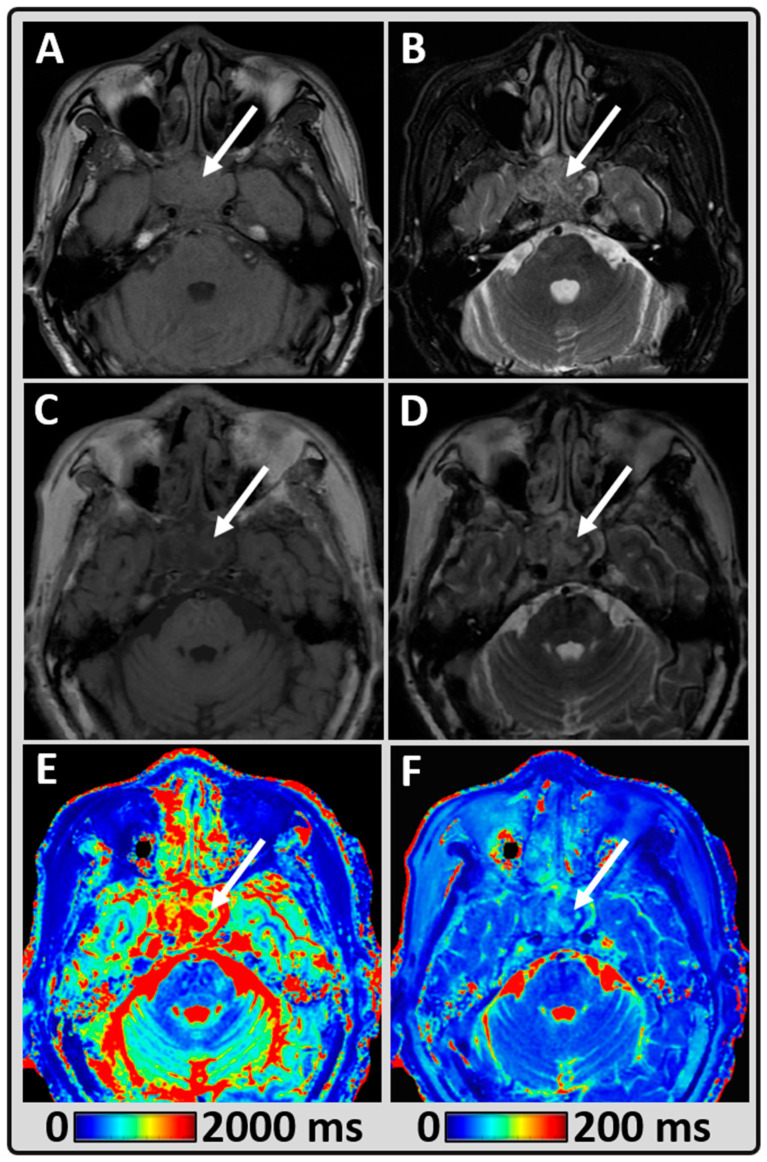
Representative MRI data from a 54-year-old male patient with white arrow pointing at recurrent nasopharyngeal cancer (post radiation therapy) extended to the sphenoid sinus. (**A**,**B**) Conventional qualitative pre-contrast T1w and T2w images depicting the lesion; (**C,D**) Synthetically generated T1w and T2w images using MAGiC; (**E**,**F**) MAGiC-generated quantitative T1 and T2 maps.

Figure 4 shows the third representative HN patient data and shows a heterogeneously enhancing mass, centered at the sphenoid sinus, extending into the upper and posterior nasopharynx. The mean T1 and T2 values measured within the tumor were 1875 ms and 87 ms, respectively. The qualitative diagnostic rating scores for the conventional T1w and T2w images were excellent and good (rated five and four), respectively. The synthetic T1w and T2w were acceptable and good (rated three and four), respectively, demonstrating their value in rapid imaging protocols.

Table 2 summarizes the HN patient cohort’s T1 and T2 values for normal-appearing masseter muscle and tumors. Mean T1 and T2 values for the masseter muscle were 880 ms and 46 ms, respectively. In the untreated group, the mean T1 and T2 values in the tumors were 1745 ms and 107 ms. Similarly, the treated group’s mean T1 and T2 values were 1930 ms and 77 ms. The T1 and T2 values measured in the tumor region between the treated and untreated groups showed no significant difference (*p*-value > 0.05). 

Considering all of the image contrast views, 64% and 93% of the synthetically generated T1w and T2w images were rated as acceptable. In comparison, 100% and 93% of the conventional clinical T1w and T2w images were rated as acceptable (Table 3).

Table 4 summarizes the legibility and morphologic features of six anatomical HN regions. The T2w images from the MAGiC method were rated comparable to the conventional T2w images, while the MAGiC T1w images were rated lower to the conventional T1w images. A few anatomical structures of interest were marked as not available (N/A) because they were not covered within the FOV (either in the conventional or MAGiC imaging) or not present (surgically resected). 

## 4. Discussion

This present study explored for the first time the utility of MAGiC in the head and neck (HN) region, which offers quantitative relaxometry metric values and qualitative contrast weighted images. Measuring the relaxation times allows assessing the subtle changes in tissue pathophysiology [34,35,36]. The clinical applicability of relaxometry mapping has been limited by the long acquisition or post-processing times for HN imaging. However, this limitation was overcome by introducing a new rapid synthetic MRI, which enables the simultaneous generation of quantitative T1 and T2 maps and qualitative contrast weighted images within a single scan [24,37]. In this exploratory study, the T1 and T2 values were quantified for normal-appearing masseter muscle and tumors in the HN region for both untreated and treated patients (not a longitudinal study). The quantitative T1 and T2 values were consistent with the previously reported values by Watanabe et al. for the masseter muscle [14]. Their study reported that the T1 (around 850 ms) and T2 (around 50 ms) values of the masseter muscle are not dependent on age or gender, as the other facial muscle values were varied. Our study reported no significant difference for both the T1 and T2 values measured in the tumor regions between the untreated and treated groups. This need to be further validated in a larger patient cohort. The MAGiC-generated and conventional T2w images were rated acceptable (93% for both). The acceptable rating for the MAGiC-generated T1w images and conventional images were 64% and 100%, respectively. The main advantage of this rapid method could be in improving patient throughput by reducing the scanning time for HN imaging. The synthetic MRI-derived T1 and T2 mapping may be useful for HN tumor characterization, which comprises a diverse group with a wide range of histological types. A study by Du et al. for the early prediction of the pathological response to neoadjuvant chemotherapy in breast cancer patients (longitudinal study) reported a decrease in both the T1 and T2 values after the treatment [38]. In a recently published study, we reported higher T1 and T2 values in a treated brain metastases (BM) patients’ group compared to an untreated group [39]. We observed a similar trend here in this HN study as well. In both of the studies (BM and HN), the relaxometry values were obtained at a single time point, not longitudinally. 

In the past, the long acquisition time of conventional T1 and T2 mapping methods was a barrier to the clinical application of relaxometry measurements. There is limited MR relaxometry literature on HN imaging. Only one study demonstrated tissue-specific quantitative MRI relaxometry measurements and non-uniform aging patterns within different regions of facial soft tissues, including the masseter muscle [14]. Kang et al. investigated the synthetic MRI at three timepoints after gadolinium-based contrast agent injection. They concluded that there are time-dependent changes in relaxation rates R1 (R1 = 1/T1) and R2 (R2 = 1/T2) in patients with brain metastases [40]. Similarly, Blystad et al. reported a significant change (Δ) in R1 (R1 = 1/T1) after gadolinium-based contrast enhancement in the peritumoral area, illustrating infiltrative tumor growth in the non-visible peritumoral region in the brain using the same synthetic MRI method [41]. These findings suggest that synthetic MRI-generated T1 and T2 mapping could be an alternative approach for assessing contrast leakage in tissue and need to be tested in HN imaging for the different subsites. We recently reported the utility of the synthetic MRI-generated T1 and T2 metric values in characterizing brain metastases and normal-appearing brain tissues [39].

The synthetic MRI was primarily developed for the brain, and investigators have recently tested the diagnostic performance of these images [24,42,43]. Tannenbaum et al. compared MAGiC to conventional brain multi-contrast MR images from 109 subjects (46 healthy individuals and 63 pathologic cases). They concluded that the image contrast was acceptable for all of the series, except T2 FLAIR [24]. West et al. compared the synthetic T1w and T2w images to the conventional images of 32 clinical pediatric brains. They reported no significant differences in sensitivity, specificity, or accuracy for specific imaging findings involving the ventricles, cerebrospinal fluid, brain parenchyma, or vasculature [42]. Ryu et al. investigated the clinical feasibility of MAGiC as a single routine neuroimaging protocol without conventional MRI in 89 brain patients. They concluded that the overall image quality and anatomical delineation provided by synthetic brain MRI were good, with diagnostic rating scores of more than three points for all of the sequences, except for T2 FLAIR [43]. These studies reported that the overall diagnostic image quality of the MAGiC-generated images for the brain was similar in most of the imaging sequences to conventional multi-contrast MRI [24,42,43]. 

The diagnostic multi-contrast and multiplanar MR images are used in clinical practice to determine the location and extent of lesions in HN cancer patients [44]. Qualitative diagnostic image analysis of the tumors in the HN region is sometimes challenging, due to their anatomical heterogeneity and complexity [6,45]. In the present exploratory study, we tested synthetically generated qualitative images acquired for four subsites, i.e., the neck, nasal, parotid, and nasopharynx, using fixed TR = 500 ms, TE = 10 ms, for the T1w images, and TR = 4500 ms, and TE = 100 ms for T2w images. The difference in the rating for the T1w images from synthetic and conventional images could be attributed to the fixed TR/TE combination. This study can be the building block for future studies that fine-tune the parameter combination based on the specific HN subsites.

As reviewed by the neuroradiologist, the legibility (or visibility of margins and structures associated with key anatomic/morphologic features) of the anatomies, agreed by 93% for the sternocleidomastoid muscle seen on both the synthetic and conventional T2w images. For example, in the nasopharynx, the legibility of the synthetic T1w and T2w images were 72% and 79%, whereas those of the conventional were 93% and 86%, respectively. As HN imaging comprises many subsites, each protocol must be vigorously tested before inclusion in a rapid, clinical diagnostic MRI exam.

Our present exploratory study had a few limitations. The sample size is small as it follows institutional guidelines for the testing of a new MRI research prototype sequence (such as MAGiC) that is not yet part of clinical HN diagnostic imaging. HN MR imaging has multiple protocols depending on the subsite. Here, we included MAGiC as a research imaging sequence in four subsites, a prerequisite before using the new method clinically for the oncological endpoints. The initial results need to be validated in a larger cohort, and the testing of the MAGiC method needs to be further extended to HN subsites prior to inclusion in rapid HN MR imaging protocols. Herein, we focused only on the simultaneous measurement of the quantitative T1 and T2 metric values and the qualitative T1w and T2w images. Future studies could be extended to additional contrast images. For example, STIR images are part of the neck imaging protocols for patients with dental implants. MAGiC, in combination with other functional imaging such as diffusion weighted, and dynamic contrast-enhanced MRI, may provide better insight into tumor physiology. 

## 5. Conclusions

In conclusion, the benefit of MAGiC in HN imaging is twofold, providing relaxometry maps in a clinically feasible time and the ability to generate a different combination of contrast images in a single acquisition. The present exploratory study reports the quantitative T1 and T2 values for normal-appearing masseter muscles and HN tumors. The diagnostic image quality of the synthetically generated images was clinically acceptable for the T2w images, and the T1w images require further improvement. This need to be further validated in a larger patient cohort.

## Figures and Tables

**Table 1:** Patient Characteristics.

Characteristics	Value
Total Patients	14
Demographics	
Median age (y)	61
Age range (y)	29–83
Male/Female	8/6
Location of tumor in HN region	
Major Salivary Gland	5
Sinonasal	3
Larynx	1
Cheek	1
Cutaneous	1
Thyroid	1
Skull Base	1
Perineural (pterygopalatine fossa)	1
Treatment timepoint	
Pre-treatment	3
Post-treatment	11
Therapy types	
CRT	2
RT and Surgery	3
CRT and Surgery	2
RT alone	1
Proton therapy alone	1
Surgery alone	2

**Table 2:** T1 and T2 values from normal-appearing masseter muscle and tumor regions.

Tissue		T1 (in ms)			T2 (in ms)		
		Range [min, max]	Mean ± SD	Median	Range [min, max]	Mean ± SD	Median
**Normal appearing** **Masseter Muscle (MM)** **(*n* = 14)**		[729, 950]	880 ± 52	876	[42, 52]	46 ± 3	45
**Tumor**	**Untreated (*n* = 2)**	[1455, 2035]	1745 ± 410	1745	[64, 150]	107 ± 61	107
	**Treated** **(*n* = 8)**	[1227, 2751]	1930 ± 422	1905	[57, 92]	77 ± 13	75

**Table 3:** Diagnostic image quality rating of synthetic and conventional T1w and T2w images.

Diagnostic Quality of the Image (*n* = 14)	Synthetic (MAGiC)		Conventional	
	T1w	T2w	T1w	T2w
**Acceptable (rated 5, 4, and 3)**	**9 (64%)**	**13 (93%)**	**14 (100%)**	**13 (93%)**
Excellent (rated 5)	0 (0%)	1 (7%)	2 (14%)	0 (0%)
Good (rated 4)	1 (7%)	6 (43%)	8 (57%)	5 (36%)
Acceptable (rated 3)	8 (57%)	6 (43%)	4 (29%)	8 (57%)
**Unacceptable (rated 2 and 1)**	5 (36%)	1 (7%)	0 (0%)	1 (7%)
Poor (rated 2)	4 (29%)	1 (7%)	0 (0%)	1 (7%)
Unacceptable (rated 1)	1 (7%)	0 (0%)	0 (0%)	0 (0%)

**Table 4:** Legibility of anatomic and morphologic features.

Legibility (*n* = 14)		SG *n* (%)		NP *n* (%)		BoT *n* (%)		SCM *n* (%)		Mandible *n* (%)		Glottis *n* (%)	
		T1w	T2w	T1w	T2w	T1w	T2w	T1w	T2w	T1w	T2w	T1w	T2w
**Legible**	**Conventional**	10 (72)	8 (57)	13 (93)	12 (86)	5 (36)	3 (21)	13 (93)	13 (93)	11 (79)	10 (71)	3 (21)	3 (21)
	**MAGiC**	3 (21)	7 (50)	10 (72)	11 (79)	2 (14)	8 (59)	10 (72)	13 (93)	6 (44)	9 (64)	2 (14)	2 (14)
**Illegible**	**Conventional**	0 (0)	2 (14)	0 (0)	1 (7)	6 (44)	8 (59)	0 (0)	0 (0)	0 (0)	1 (7)	1 (7)	1 (7)
	**MAGiC**	7 (50)	3 (21)	3 (21)	2 (14)	9 (66)	3 (21)	3 (21)	0 (0)	5 (36)	2 (14)	2 (13)	2 (13)
**N/A**	**Conventional**	4 (28)		1 (7)		3 (20)		1 (7)		3 (20)		10 (72)	
	**MAGiC**	4 (28)		1 (7)		3 (20)		1 (7)		3 (20)		10 (72)	

Note: SG: Submandibular gland; NP: Nasopharynx; BoT: Base of tongue; SCM: Sternocleidomastoid muscle.

## Data Availability

The data presented in this study will be provided upon reasonable request.
